# Associations between youth suicide rates and state school personnel suicide prevention training requirements

**DOI:** 10.1016/j.pmedr.2024.102768

**Published:** 2024-05-21

**Authors:** Meghan L. Shah-Hartman, Katie E. Greenawalt, Eric W. Schaefer, Deepa L. Sekhar

**Affiliations:** a Department of Pediatrics, Penn State College of Medicine, 90 Hope Drive A145 Hershey, PA 17033, United States; b Department of Public Health Sciences, Penn State College of Medicine, 700 HMC Crescent Road Hershey, PA 17033, United States

**Keywords:** Youth suicide prevention training, School personnel training, Suicide prevention legislation, Educator suicide prevention training, Youth suicide rate, United states

## Abstract

**Objective:**

As youth spend the majority of their time in school, school personnel suicide prevention training has gained support as an approach to mitigate rising youth suicide rates. This study examined associations between state school personnel suicide prevention training requirements (i.e., mandatory/non-mandatory and annual/not annual), year of legislation enactment (2013 or earlier/2014 or later), and changes in youth suicide rates by state from 2007-09 to 2016–18.

**Methods:**

School personnel suicide prevention training requirement data were collected through state-specific Department of Education websites while state-specific youth suicide rates were collected from the Center for Disease Control and Prevention’s 2020 National Vital Statistics Report. Data were analyzed using a mixed effects linear regression model.

**Results:**

Training is mandated in 40/50 (80 %) states; 19/50 (38 %) require annual training. All states demonstrated increases in youth suicide rates from 2007-09 to 2016–18 (mean increase 3.9/100,000 [sd = 1.8]), but the change did not differ significantly by state requirements for mandatory (p = 0.44) or annual (p = 0.70) training, nor for year of enactment of legislation (p = 0.45).

**Conclusions:**

School personnel suicide prevention training requirements were not associated with changes in the youth suicide rate, though study results are limited in that data was not available on how successfully schools implemented the required trainings.

## Highlights

• Youth suicide rates are on the rise.
• No significant relationship between training and suicide rates identified.
• School personnel suicide prevention training may need reform.
• A broad spectrum of adults may play an important role in youth suicide prevention.

## 1 Introduction

The United States (US) is facing a considerable rise in youth suicides. Between 2007–2018, the suicide rate among persons aged 10–24 in the US increased by 57.4 %, with significant increases in 42/50 states (Curtin, 2020). As of 2021, suicide was the third leading cause of death among US adolescents (Gaylor et al., 2023).

Given that youth spend the majority of their time at school (U.S. Department of Education. Public School Data File:, 2024), school personnel, often termed ‘gatekeepers’ in youth suicide prevention training, play a crucial role in recognizing and responding to early signs of suicide (Stickl Haugen et al., 2022, Mo et al., 2018, Hatton et al., 2017). While participation of school personnel in suicide prevention training has been positively associated with their level of self-efficacy to engage in suicide prevention (Stickl Haugen et al., 2022, Mo et al., 2018, McKoin Owens et al., 2022, Wyman et al., 2008), systematic reviews demonstrate inconsistent results with respect to identification and referral behavior (Mo et al., 2018, Torok et al., 2019). It also remains unclear if state suicide prevention training requirements are associated with changes in youth suicide rates by state over time.

The study aim was to identify associations between state school personnel suicide prevention training requirements (specifically mandatory/non-mandatory and annual/not annual), year of enactment of suicide prevention training legislation (2013 or earlier/2014 or later), and changes in youth suicide rates from 2007-09 to 2016–18.

## 2 Methods

This study received a non-human subjects research determination by the Penn State College of Medicine Institutional Review Board on August 25th, 2023. This study was based on publicly available data and is thus exempt form ethical compliance.

### 2.1 Youth suicide rates

Youth suicide rates by state were based on the Center for Disease Control and Prevention’s (CDC) 2020 National Vital Statistics Report (Curtin, 2020). These data include the number of deaths due to suicide among persons aged 10–24 per 100,000 population. This analysis used the three-year average suicide rates from 2007-2009 and 2016–2018 by state (Curtin, 2020).

### 2.2 Search strategy for school personnel suicide prevention training policies

State Department of Education websites and legislation were reviewed to obtain data on state-specific requirements for school personnel suicide prevention training. Data were collected for all 50 states (excluding Washington D.C. and Puerto Rico). When data remained inaccessible, two e-mail attempts were made to contact state representatives, with a follow-up telephone call if needed. If state representatives were unreachable, the American Foundation for Suicide Prevention (AFSP) website was utilized to reach AFSP state-specific representatives. Four total contact attempts were made to obtain complete data between 9/4/23–11/2/23.

### 2.3 Classification of data and statistical analysis

The suicide prevention training data was categorized by state, and included whether training was mandatory (yes/no), training frequency (annual/not annual), and year of enacted legislation (2013 or earlier/2014 or later, selected as the 2007–2018 timeframe midpoint). A mixed effects linear regression model examined associations between mandatory training requirements and changes to the youth suicide rate over time. The model included fixed effects for time (2007–09 vs. 2016–18), an indicator whether training was mandatory (yes/no), the interaction between time and mandatory training, and a random effect for state to account for the correlation over time within each state. The interaction term represents the difference in the change over time (slope) between states that mandated training versus states that did not, and was the parameter of interest in the analysis. The same framework was used to examine training frequency and year of enacted legislation.

## 3 Results

Data was obtained for all 50 states, though nine required contacting state-specific Department of Education and AFSP representatives to confirm legislation details. School personnel suicide prevention training is currently mandated in 40/50 (80 %) states with 19/50 (38 %) stipulating annual training (Supplementary Table 1). Other states require training every 2–5 years. Legislation enactment spanned 2006–2023, and 18/50 (36 %) states enacted legislation in 2013 or prior. All 50 states demonstrated a significant increase in youth suicide rates (per 100,000) from 2007-09 to 2016–18 (mean increase 3.9 percentage points [s.d. = 1.8]).

**Fig. 1:**
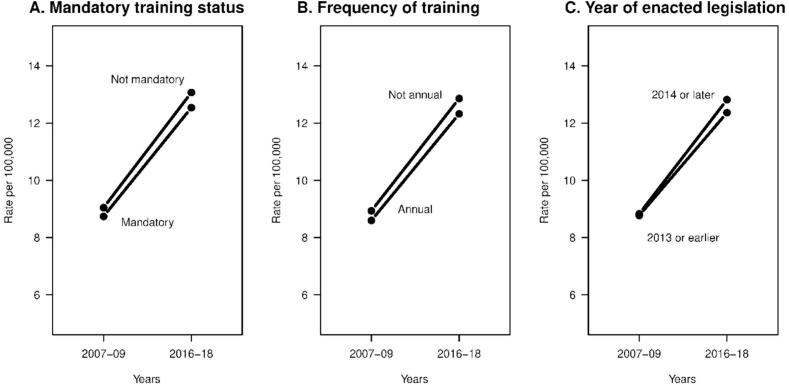
Visual depiction of estimated United States youth suicide rate^1^ from 2007-09 to 2016–18 by school personnel suicide prevention training requirements from fitted regression models. ^1^ Deaths due to suicide among persons aged 10–24 per 100,000 population.

Changes in the youth suicide rates from 2007-09 to 2016–18 were not significantly associated with state training requirements or year of legislation enactment in the fitted statistical models (Table 1). States with mandatory training had an increase in the youth suicide rate (per 100,000) of 3.8 versus 4.2 for states without mandatory training, a difference of 0.4 (interaction term p = 0.44). Differences in rates for training frequency (0.2 per 100,000; 3.7 for annual versus 3.9 for not annual; interaction p = 0.70) and for year of enacted legislation (0.4 per 100,000; 3.6 for 2013 or prior versus 4.0 for 2014 or later; interaction term p = 0.45) were not statistically significant (Fig. 1).

**Table 1:** Fitted regression models for changes in United States youth suicide rate^1^ from 2007-2009 and 2016–2018 based on school personnel suicide prevention training requirements.

Variable	Parameter	Estimate (95 % CI)	p-value^2^
Mandatory	Intercept	9.3 (6.5, 12.1)	<0.001
training	Mandatory training (in 2007–09)		
	Yes	−0.6 (−3.8, 2.5)	0.70
	No (ref)	0	
	Change from 2007-09 to 2016–18 (interaction term)		0.44
	Mandatory training	3.8 (3.2, 4.3)	<0.001
	Not mandatory training	4.2 (3.1, 5.3)	<0.001
Annual	Intercept	8.9 (7.3, 10.5)	<0.001
training	Frequency of training (in 2007–09)		
	Annual	−0.3 (−2.9, 2.3)	0.80
	Not annual (ref)	0	
	Change from 2007-09 to 2016–18 (interaction term)		0.70
	Annual training	3.7 (2.9, 4.5)	<0.001
	Not annual training	3.9 (3.3, 4.6)	<0.001
Year of	Intercept	8.8 (6.7, 10.9)	<0.001
legislation	Year of legislation (in 2007–09)		
	2014 or later	0.1 (−2.6, 2.7)	0.97
	2013 or earlier (ref)	0	
	Change from 2007-09 to 2016–18 (interaction term)		0.45
	2014 or later	4.0 (3.4, 4.6)	<0.001
	2013 or earlier	3.6 (2.8, 4.4)	<0.001

1 Deaths due to suicide among persons aged 10–24 per 100,000 population.

2 Parameters in the mixed effects models were estimated using restricted maximum likelihood (REML), with p-values being derived from t-tests using Satterthwaite’s method. Changes from 2007-09 to 2016–18 were constructed from contrasts of estimated model parameters using the same t-tests.

## 4 Discussion

This analysis did not demonstrate an association between state school personnel suicide prevention training requirements, year of legislation enactment, and changes in youth suicide rates from 2007-09 to 2016–18. However, the study results should not suggest that school personnel suicide prevention training is not effective; rather, there may be additional factors impacting school personnel application of suicide prevention training and youth suicide rates (Stickl Haugen et al., 2022, Torok et al., 2019, Risk and Protective Factors. Centers for Disease Control and Prevention. November 2, 2022, Kõlves et al., 2017, Bevilacqua, 2023).

Research demonstrates that school personnel who experience student death by suicide suffer significant distress and demonstrate significantly higher reluctance to engage as gatekeepers in suicide prevention compared to those who have not had this experience (Stickl Haugen et al., 2022, Kõlves et al., 2017). Thus, school personnel in states with higher youth suicide rates may require additional support to engage with suicide prevention training. In addition, school personnel required to participate in training varied considerably by state. Some required all personnel (teachers, counselors, administration, custodial, food services, coaches and volunteers), and others only counselors. Therefore, our findings raise questions regarding extending training to a broader spectrum of the adults (e.g., all school staff and even parents, clergy) that routinely influence today’s youth (Torok et al., 2019).

Data collection was complicated by legislative changes during the study time period, and finer details were challenging to categorize. For example, from 2017 to 2018 Indiana updated training guidelines from recommended to mandated; classified as mandatory in the analysis (Indiana Department of Education. Suicide Prevention Training, 2023). Rhode Island mandates training for all school personnel without specifying training duration (Rhode Island General Assembly, 2024), while Pennsylvania requires four hours of training every five years (Pennsylvania Department of Education, 2024). Study findings were also limited by the selected timepoints (2007–09 to 2016–18), and the inability to adjust for inherent differences in youth demographics by state**.** Finally, this study focused solely on suicide prevention training requirements, but did not address how school systems in each state implemented training requirements, which further impacts program effectiveness.

## 5 Conclusion

In summary, the study did not find an association between state school personnel suicide prevention training requirements and changes in youth suicide rates. Variation in state suicide prevention training requirements for school personnel highlights an opportunity to continue to track the relationship between suicide prevention policies and youth suicide rates, allowing researchers to identify interventions that best support youth mental wellness.

## CRediT authorship contribution statement

**Meghan L. Shah-Hartman:** Writing – review & editing, Writing – original draft, Project administration, Methodology, Investigation, Conceptualization. **Katie E. Greenawalt:** Writing – review & editing, Writing – original draft, Project administration, Methodology, Conceptualization. **Eric W. Schaefer:** Writing – original draft, Project administration, Methodology, Formal analysis, Conceptualization. **Deepa L. Sekhar:** Writing – review & editing, Writing – original draft, Project administration, Methodology, Conceptualization.

## Declaration of competing interest

The authors declare that they have no known competing financial interests or personal relationships that could have appeared to influence the work reported in this paper.

## Data Availability

The data have been shared through Supplementary Table 1.
